# Discovery of Anti-Aging Effects of Wheat Bran Extract in a D-Galactose-Induced Rat Model of Oxidative Stress

**DOI:** 10.3390/nu17182954

**Published:** 2025-09-13

**Authors:** Kaori Kobayashi, Keshari Sudasinghe, Ryan Bender, Md Suzauddula, Cheng Li, Cen Wu, Yonghui Li, Weiqun Wang

**Affiliations:** 1 Department of Food Nutrition Dietetics and Health, Kansas State University, Manhattan, KS 66506, USA; kobakaori@ksu.edu (K.K.); rdbender@ksu.edu (R.B.); suza@ksu.edu (M.S.); 2 Department of Anatomy and Physiology, Kansas State University, Manhattan, KS 66506, USA; keshari93@ksu.edu; 3 Department of Grain Science and Industry, Kansas State University, Manhattan, KS 66506, USA; chengli@ksu.edu; 4 Department of Statistics, Kansas State University, Manhattan, KS 66506, USA; wucen@ksu.edu

**Keywords:** wheat bran extracts, D-galactose, oxidative stress, mitochondrial dysfunction, superoxide dismutase, senescence-associated β-galactosidase

## Abstract

**Background/Objectives:** Wheat bran is known for its anti-aging effects, primarily due to its antioxidant properties. Our previous study identified novel antioxidants in wheat bran (xylo-oligosaccharides and protein hydrolysates) using an innovative extraction method. However, the anti-aging potential of these wheat bran extracts (WBEs) remains unclear. **Methods:** This study evaluated the anti-aging effects of WBE in a D-galactose-induced aging model using Wistar rats. Animals were divided into four groups: (1) saline-injected control, (2) D-galactose-injected control, (3) D-galactose + 5% WBE, and (4) D-galactose + 10% WBE. After six weeks, body weight, food intake, body fat percentage, erythrocyte superoxide dismutase (SOD) activity, and liver senescence-associated β-galactosidase (SA-β-gal) levels were assessed. **Results:** D-galactose significantly reduced food intake in positive control 87 ± 21%/weekly (negative control; *p* < 0.05, 107 ± 20%/weekly for 10%WBE; *p* < 0.01. Body fat percentage (positive control: 84 ± 19% vs. 5% WBE: 110 ± 20%, *p* < 0.05 in 100% convert). It also lowered erythrocyte SOD activity; 68.6 ± 9%, *p* < 0.01 in 100% conversion). WBE supplementation restored SOD activity in a dose-dependent manner (5% WBE: 32,479 ± 12,773 U/mL; 10% WBE: 42,368 ± 20,281 U/mL. Although D-galactose did not elevate significantly SA-β-gal activity in the liver, WBE supplementation still led to a dose-dependent reduction in baseline SA-β-gal levels (294 ± 84 nmol/min/mg protein vs. 5% WBE: 181 ± 65 nmol/min/mg protein, and 10% WBE: 146 ± 40 nmol/min/mg protein. *p* < 0.001). No significant group differences were found in hepatic SOD2, catalase (liver and skin), or telomerase reverse transcriptase expression. **Conclusions:** These findings suggest that wheat bran extracts mitigate D-galactose-induced oxidative stress in circulation, indicating potential anti-aging benefits. However, their effects at the tissue level remain inconclusive. Further studies are needed to explore molecular mechanisms and refine intervention duration.

## 1. Introduction

Aging is a multifactorial biological process characterized by progressive physiological decline, increased susceptibility to chronic diseases, and impaired cellular repair mechanisms [1,2]. It is closely associated with oxidative stress, mitochondrial dysfunction, and telomere attrition, which collectively drive tissue degeneration and cellular senescence [3,4,5]. With the rapidly aging global population, the development of safe and effective strategies to delay or mitigate aging-related disorders has become a pressing research priority [6].

Classical biomarkers of aging, including superoxide dismutase (SOD), catalase, senescence-associated β-galactosidase (SA-β-gal), and telomerase reverse transcriptase (TERT), have provided important insights into redox homeostasis, senescence, and telomere maintenance [7,8,9]. While these markers remain essential for establishing aging-related changes, their roles are already well established [10,11,12,13]. Consequently, current gerontological research is increasingly focuses on identifying novel dietary bioactive compounds that can modulate these pathways and provide practical applications for healthy aging.

Wheat bran, a by-product of the milling process, has attracted growing interest due to its abundance of bioactive compounds with antioxidant, anti-inflammatory, and potential anti-aging properties [14,15]. Our recent work has shown that wheat bran extracts (WBE) contain functional components such as xylo-oligosaccharides and protein hydrolysates, which exhibit strong free radical-scavenging activity, enzyme inhibitory effects, and potential utility in both food and cosmetic formulations [16]. Despite this evidence, the direct role of wheat bran-derived antioxidants in modulating aging processes in vivo remains insufficiently investigated. Most prior studies have focused on general health benefits, leaving a significant gap regarding their mechanistic effects on molecular markers of aging [17].

Building on our group’s prior findings on wheat bran antioxidants, the present study investigates their effects in a D-galactose-induced aging model [17]. Specifically, we aim to determine whether these novel wheat bran compounds (xylo-oligosaccharides and protein hydrolysates) can attenuate oxidative stress, modulate SA-β-gal activity, and support telomere stability, thereby providing a natural intervention against accelerated aging. Growing evidence suggests that dietary antioxidants may mitigate age-related oxidative damage and cellular senescence. WBE, rich in phenolic compounds, has demonstrated antioxidative potential in vitro [15]; however, it’s in vivo efficacy remains poorly characterized. Therefore, we hypothesize that oral administration of WBE will attenuate D-galactose-induced oxidative stress and cellular senescence in a rat model of accelerated aging, potentially through modulation of endogenous antioxidant defense mechanisms. This is a prespecified, confirmatory study.

## 2. Materials and Methods

### 2.1. Wheat Bran Extract (WBE) Preparation and Handling

Hard red winter wheat bran (protein content: 16.8%) was sourced from Hal Ross Flour Mill (Manhattan, KS, USA) [16]. The wheat bran underwent grinding and a series of processing steps to extract xylo-oligosaccharides and protein hydrolysates as novel antioxidants identified in our recent studies [14,15]. These wheat bran extracts were combined with the insoluble residues and subsequently incorporated into experimental diets to evaluate their efficacy in mitigating oxidative stress and promoting healthy aging. These wheat bran extract (WBE) diets, along with the basal AIN-93G diet, were formulated and pelleted by Envigo Teklad (Madison, WI, USA). The WBE diets were designed to match the basal AIN-93G diet in terms of fat, protein, fiber, essential micronutrient levels, and total caloric content, with carbohydrate composition modified to include either 5% or 10% WBE (Table 1). The WBE samples in this study were received and processed within approximately three months of preparation; however, no formal stability assessment was performed. Future studies should incorporate dedicated stability evaluations to strengthen the characterization of WBE and its bioactive properties. 

### 2.2. Experimental Approach

This study employed a D-galactose-induced aging model in Wistar rats to evaluate the anti-aging effects of wheat bran extract (WBE), following established protocols for oxidative stress and senescence induction [18,19,20]. The study protocol was not registered. All procedures were approved by the Institutional Animal Care and Use Committee of Kansas State University and conducted in accordance with established guidelines. Criteria for early removal or euthanasia included significant weight loss (>20%), persistent lethargy, inability to access food or water, severe wounds or infections, or any signs of unrelievable suffering, as specified in the IACUC-approved protocol.

Forty female Wistar rats (8 weeks old, weighing 193 ± 20 g) were purchased from Charles River Laboratories (O’Fallon, MO, USA) and housed in a pathogen-free facility under controlled environmental conditions. The sample size was determined based on intra- and inter-group variation reported in published studies, using power calculation according to statistical principles. The required sample size for each treatment group to detect a prespecified minimum difference (D) between two groups with power ≥1 − β (0 < β < 1) is given by *n* = (Zα/2 + Zβ)^2^ × V/D^2^, where Zp represents the (1 − p)100 percentile of the standard normal distribution and V is the variance of the comparison. Based on a previous publication [11], the minimal number of rats required per group was approximately 10. The animals were maintained on a standard 12:12-h light-dark cycle and provided with a standard AIN-93G pellet diet and water ad libitum during a one-week acclimatization period. After acclimatization, rats were randomly allocated into four experimental groups (*n* = 10 per group) using a computer-based random number generator. The experimental groups were as follows: (1) Negative Control: Saline injection + AIN-93G diet; (2) Positive Control: D-galactose injection + AIN-93G diet; (3) 5% WBE: D-galactose injection + 5% WBE diet; and (4) 10% WBE: D-galactose injection + 10% WBE diet.

Female rats were used in this study because the project focused on developing antioxidant compounds from wheat bran for potential applications in anti-aging cosmeceuticals and nutraceuticals, which are often target toward female consumers.

D-galactose was administered intraperitoneally at a dose of 200 mg/kg/day, following established protocols that reliably induce aging-like phenotypes [21]. To minimize animal discomfort, appropriate restraint techniques were used, all injections were performed by trained personnel, and animals were monitored for signs of distress after each daily injection. Animal accessed diet and water ad libitum Animals were monitored and recorded daily for signs of pain or distress by trained personnel. Body weight and food intake were recorded weekly throughout the six-week intervention. At study completion, body fat percentage was assessed using PIXImus Densitometry (GE Healthcare, Chicago, IL, USA), a validated method for small animal body composition analysis.

### 2.3. Sample Collection and Biochemical Analyses

All animals completed the study, and all collected data were included in the analysis. All experimental groups contained 10 animals. Blood, liver, and skin tissues were collected post-sacrifice. Erythrocyte SOD activity was quantified using the WST-based SOD Assay Kit (Dojindo, Kumamoto, Japan), a photometric method validated for sensitivity and reproducibility in antioxidant studies. Protein expression of catalase, SOD2, and TERT in liver and skin tissues was assessed via Jess automated western blotting (Bio-Techne, Minneapolis, MN, USA), following protocols adapted from previous antioxidant and aging biomarker research.

SA-β-gal activity in liver tissues was measured using a fluorescence-based β-galactosidase assay (Abcam, Waltham, MA, USA), consistent with established senescence detection protocols.

### 2.4. Analysis of SOD in Erythrocytes

Superoxide dismutase is a key antioxidant enzyme that plays a vital role in protecting cells from oxidative stress [22]. To quantify SOD activity in erythrocytes, a photometric analysis was conducted using the SOD Assay Kit—WST (Dojindo Molecular Technologies, Inc., Rockville, MD, USA). The assay was performed following the manufacturer’s protocol. First, all necessary reagents, including the WST-1 substrate, enzyme standards, and buffers, were prepared. Erythrocyte samples were then collected, processed, and diluted as per the assay requirements. The prepared samples and enzyme standards were added to a reaction mixture containing the WST-1 substrate in a 96-well microplate. Absorbance was measured at 450 nm before and after the reaction to monitor changes in optical density. The difference in absorbance values was used to calculate SOD activity, which was determined by comparing sample readings to a standard curve generated using known concentrations of SOD enzyme. To ensure accuracy and reproducibility, all experiments were conducted in triplicate, and results were expressed as mean values with standard deviations.

### 2.5. Analysis of Catalase, SOD2, and TERT in Skin and Liver Tissues

Protein expression levels of catalase, SOD2, and TERT in posterior skin and liver tissues were analyzed via Jess automated western blotting (Bio-techne, Minneapolis, MN, USA). Total protein was extracted from tissues using RIPA Lysis Buffer (Sigma-Aldrich, St. Louis, MO, USA) according to the manufacturer’s instructions. Protein concentrations were quantified using the Qubit 4 Fluorometer with the Protein Broad Range Assay (Thermo Fisher Scientific Inc., Waltham, MA, USA). For sample preparation, 85 mg of back skin tissue and 20–40 mg of randomly collected liver tissue were incubated with RIPA buffer (200 µL for skin and 400 µL for liver) containing a protease inhibitor on ice for 20 min. The tissues were then homogenized using a Dounce pestle, with skin homogenized for >100 s (5–8 passes) and liver for 90 s (4 passes). Following homogenization, the samples were incubated on a tube rack for 20 min. Skin homogenates were centrifuged at 13,000 RPM for 3 min, while liver homogenates were centrifuged at 14,000 RPM for 10 min at 4 °C. Supernatants were collected, and the lipid layer in liver samples was removed and discarded. Protein concentrations in the lysates were equalized before western blotting, and homogenized samples were stored at −80 °C until analysis. Western blotting was performed using the Jess Protein Simple automated western blotting system (Bio-Techne, Minneapolis, MN, USA). Target proteins included catalase (Abcam, Waltham, MA, USA), SOD2 (Novus, Littleton, CO, USA), and TERT (Thermo Fisher Scientific Inc., Waltham, MA, USA). Protein expression data were quantified using the area under the curve (AUC) values generated by Compass software (Compass Engineering, Cincinnati, OH, USA).

### 2.6. Analysis of β-Galactosidase

The SA-β-gal assay was conducted using the beta Galactosidase Assay Kit according to the manufacturer’s instructions (Abcam, Waltham, MA, USA). Briefly, frozen liver sections were prepared, washed, and stained with β-galactosidase staining solution and incubated at 37 °C in a dry incubator. Fluorescence (Ex/Em = 480/530 nm) was measured using the Multiskan™ GO microplate spectrophotometer (Thermo Fisher Scientific, MA). Fluorescence values were applied to a standard curve to derive absorbance measurements. Percent inhibition is determined by the following formula:

$$\mathrm{Inhibition}\;\mathrm{rate}\;(\%)\;=\;\frac{\left(S1-S3\right)-(SS-S2)}{\left(S1-S3\right)}\;\times \;100$$

where: *S*1: the slope of blank 1; *S*2: the slope of blank 2; *S*3: the slope of blank 3; and *SS*: the slope of the sample.

### 2.7. Statistical Analysis

Data were expressed as means ± SEM. Statistical analyses were conducted using GraphPad Prism 8 software (GraphPad Software, Inc., San Diego, CA, USA). Comparisons between two groups were made using unpaired Student’s *t*-tests, while differences among more than two groups were analyzed using one-way ANOVA. For non-normally distributed data, as indicated by the Shapiro-Wilk test, nonparametric statistical tests, including the Kruskal-Wallis test, were applied. In addition to p-values, effect sizes (Cohen’s *d* for pairwise comparisons, η^2^ for ANOVA, and rank-biserial correlation for nonparametric tests) and 95% confidence intervals (CIs) were reported to evaluate the magnitude and precision of group differences. A *p*-value <0.05 was considered statistically significant.

In addition to *p*-values, effect sizes (Cohen’s *d* for pairwise comparisons, η^2^ for ANOVA, and rank-biserial correlation for nonparametric tests) and 95% confidence intervals (CIs) were reported to assess the magnitude and precision of group differences. For visualization, group means and standard deviations were normalized to the negative control (set as 100%). All statistical analyses, including effect size and confidence interval calculations, were performed using raw data to ensure accuracy and interpretability. A *p*-value < 0.05 was considered statistically significant.

## 3. Results

### 3.1. Change in Body Weights and Food Intake

**Figure 1:**
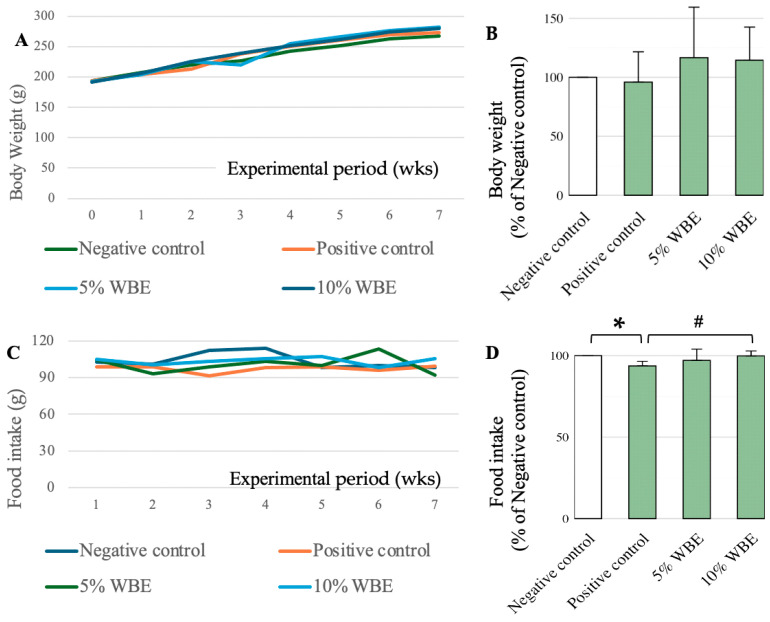
Body weight transition and food intake over the experimental period. (**A**) Body weight changes in aging rats throughout Experiment B. The rate of weight change for each rat was calculated by dividing the final week’s body weight by the initial week’s body weight and multiplying by 100. (**B**) Group-wise comparison of weight change rates. (**C**) Transition in mean daily food intake over the experimental period. (**D**) Final week’s food intake, averaged per individual, was compared across groups. The saline-injected negative control group was set as the baseline (100%), and all other values were expressed as percentages relative to this group. Data are presented as mean ± SD (*n* = 10). When normalized to the negative control group, the positive control group showed a mean of 86.7% ± 20.8%, and the 10% WBE group showed 106.6% ± 19.6%. * *p* < 0.05, ^#^ *p* < 0.01. Cohen’s *d* values and 95% confidence intervals for significant comparisons. Positive vs. Negative control: Cohen’s *d* = 1.43, 95% CI [0.62, 2.20], and vs. 10% WBE: Cohen’s *d* = 2.05, 95% CI [1.25, 2.85]. Group definitions: Negative control: Saline-injected rats, Positive control: D-galactose-injected rats, 5% WBE: D-galactose-injected rats fed a diet containing 5% wheat bran extract, 10% WBE: D-galactose-injected rats fed a diet containing 10% wheat bran extract.

Throughout the experimental period, the aging rats (positive control) and WBE treatment groups received daily D-galactose injections (200 mg/kg). Weekly weight measurements showed an average weight gain of approximately 12 g per week across all groups. However, the weight gain was not consistent throughout the experimental period, with the 5% WBE and positive control groups exhibiting fluctuations in weight gain (Figure 1A,B). The average daily food intake was approximately 14 g/rat/day, calculated based on weekly food consumption. However, food intake varied significantly among groups. The galactose-positive control group consumed significantly less food than the negative control group (*p* < 0.05, Cohen’s *d* = 1.43, 95% CI [0.62, 2.20]). In contrast, the 5% and 10% WBE supplementation groups exhibited food intake levels comparable to the negative control group, with the 10% WBE group consuming significantly more than the positive control group (*p* < 0.01, d = 2.05, 95% CI [1.25, 2.85]) (Figure 1C,D).

### 3.2. Effect of WBE Supplements on Body Fat Percentage in Aging Rats

**Figure 2:**
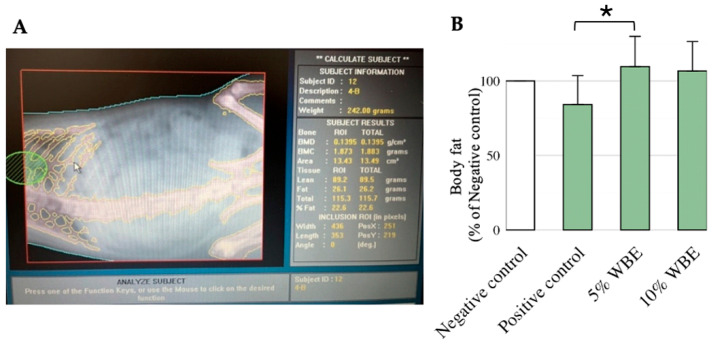
Effect of WBE treatment on body fat percentage in D-galactose-induced accelerated aging rats. (**A**) Body fat percentage measured in the 7th week at sacrifice using a Lunar Pixi densitometer. (**B**) Mean body fat percentage across all groups at the end of the experimental period. The saline-injected negative control group was set as the baseline (100%), and all other values were expressed as percentages relative to this group. Data are presented as mean ± SD (*n* = 10). When normalized to the negative control group, the positive control group showed a mean of 84.1% ± 18.7%, while the 5% WBE group showed 109.6% ± 19.8%. * *p* < 0.05 compared with the galactose-injected positive control group. Cohen’s *d* and 95% confidence interval for the comparison between the positive control and 5% WBE group: Cohen’s *d* = 1.78, 95% CI [0.91, 2.63]. Group definitions: Negative control: Saline-injected rats, Positive control: D-galactose-injected rats, 5% WBE: D-galactose-injected rats fed a diet containing 5% wheat bran extract, 10% WBE: D-galactose-injected rats fed a diet containing 10% wheat bran extract.

The negative control group exhibited an average body fat percentage of 27%. Both the 5% and 10% WBE supplementation groups maintained similar body fat percentages of approximately 30%, indicating consistent results across these groups. In contrast, the galactose-injected positive control group displayed a significantly lower body fat percentage (23%) (*p* < 0.05, Cohen’s *d* = 0.82, 95% CI [0.21, 1.39]), suggesting that D-galactose administration altered body composition (Figure 2B).

### 3.3. Effect of WBE Supplements on SOD in Aging Rats

**Figure 3:**
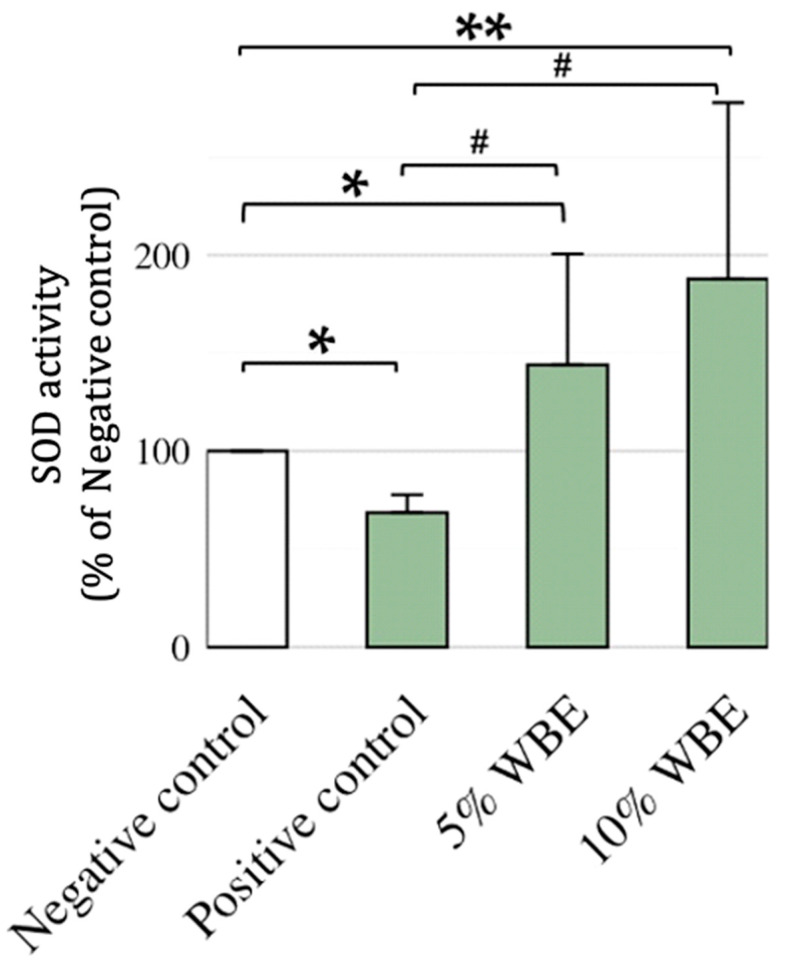
Effect of WBE treatment on SOD Activity in galactose-induced accelerated aging rats. Superoxide dismutase (SOD) activities (U/mg protein) in erythrocytes of galactose-induced aging female Wister rats treated with WBE-supplemented diets at concentrations of 5% or 10%. SOD values were determined as described in the Materials and Methods section. The saline-injected negative control group was set at 100% (baseline) and compared with each rat in all groups. Data are presented as mean ± SD (*n* = 10). When normalized to the negative control group, the positive control group showed a mean of 68.6% ± 9.0%, 5% WBE group showed 140.0% ± 67.4%, and the 10% WBE group showed 177.8% ± 89.0%. * *p* < 0.05, ** *p* < 0.01, ^#^ *p* < 0.001 compared with the saline-injected negative control group. Negative control, Saline-injected negative control group; Positive control, D-galactose-injected positive control group; 5% WBE, D-galactose-injected with 5% Wheat bran extract diets; 10% WBE, D-galactose-injected with 10% Wheat bran extract diets.

The activity of SOD in erythrocytes and liver tissue was assessed to determine the antioxidant effect of WBE supplementation. The galactose-positive control group exhibited significantly lower SOD activity in erythrocytes compared to the negative control group, indicating oxidative stress induction (Figure 3). WBE supplementation produced a dose-dependent increase in erythrocyte SOD activity, with 5% WBE elevating activity to 32,749 U/mL, and 10% WBE increasing it to 47,368 U/mL in comparison, the positive control group (G2) exhibited a significantly lower value than the negative control (Cohen’s *d* = −1.19, 95% CI [−1.93, −0.42]). Conversely, groups G3 and G4 showed markedly higher values, with large effect sizes (G3: *d* = 0.99, 95% CI [0.23, 1.72]; G4: *d* = 1.48, 95% CI [0.66, 2.28]), indicating biologically meaningful increases. The disparity between erythrocytes and the liver suggests that WBE primarily exerts its antioxidative effect in circulating blood cells rather than hepatic mitochondria. As summarized in effect, differences in oxidative stress sources, SOD isoforms, and metabolic adaptation mechanisms may explain the tissue-specific variation in response.

### 3.4. Effect of WBE Supplements of β-Galactosidase in Aging Rats

**Figure 4:**
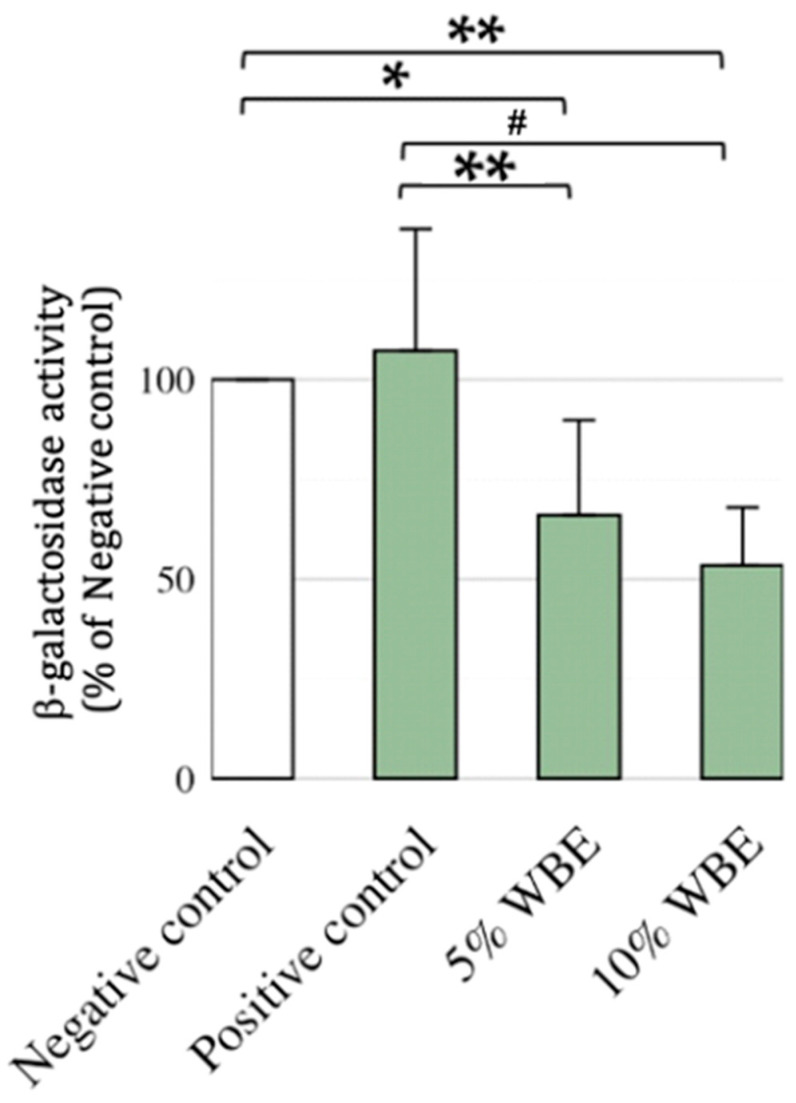
β-Galactosidase Activity in Liver. β-Galactosidase (β-gal) activity was measured in the livers of female Wistar rats subjected to D-galactose-induced accelerated aging and treated with wheat bran extract (WBE)-supplemented diets at concentrations of 5% or 10%. Enzymatic activity was assessed as described in the Materials and Methods section. The saline-injected negative control group was set as the baseline (100%), and all other values were expressed as percentages relative to this group. Data are presented as mean ± SD (*n* = 10). When normalized to the negative control group, the positive control group showed a mean of 105.1% ± 29.4%, the 5% WBE group showed 60.0% ± 4.7%, and the 10% WBE group showed 54.4% ± 12.4%. Statistical significance: * *p* < 0.01 compared with the saline-injected negative control group. ** *p* < 0.001 and ^#^ *p* < 0.0001 compared with the D-galactose-injected positive control group. Group definitions: Negative control: Saline-injected rats. Positive control: D-galactose-injected rats. 5% WBE: D-galactose-injected rats fed a diet containing 5% wheat bran extract. 10% WBE: D-galactose-injected rats fed a diet containing 10% wheat bran extract.

SA-β-gal activity was significantly elevated in the galactose-positive control group, confirming increased cellular senescence. Compared to the negative control group, the 5% WBE group showed a slightly higher value (Cohen’s *d* = 0.19, 95% CI [−0.53, 0.90]). In contrast, both the 10% WBE group and the positive control group exhibited significantly lower values, with very large negative effect sizes (5% WBE: *d* = −4.04, 95% CI [−5.30, −2.76]; 10%WBE: *d* = −3.35, 95% CI [−4.52, −2.17]), indicating strong biological suppression relative to the negative control. Notably, while D-galactose did not significantly increase SA-β-gal activity in the liver, WBE supplementation still led to a dose-dependent reduction in baseline SA-β-gal levels, suggesting a protective effect against aging-related cellular deterioration (Figure 4).

### 3.5. Protein Expression in Skin and Liver Tissues

**Figure 5:**
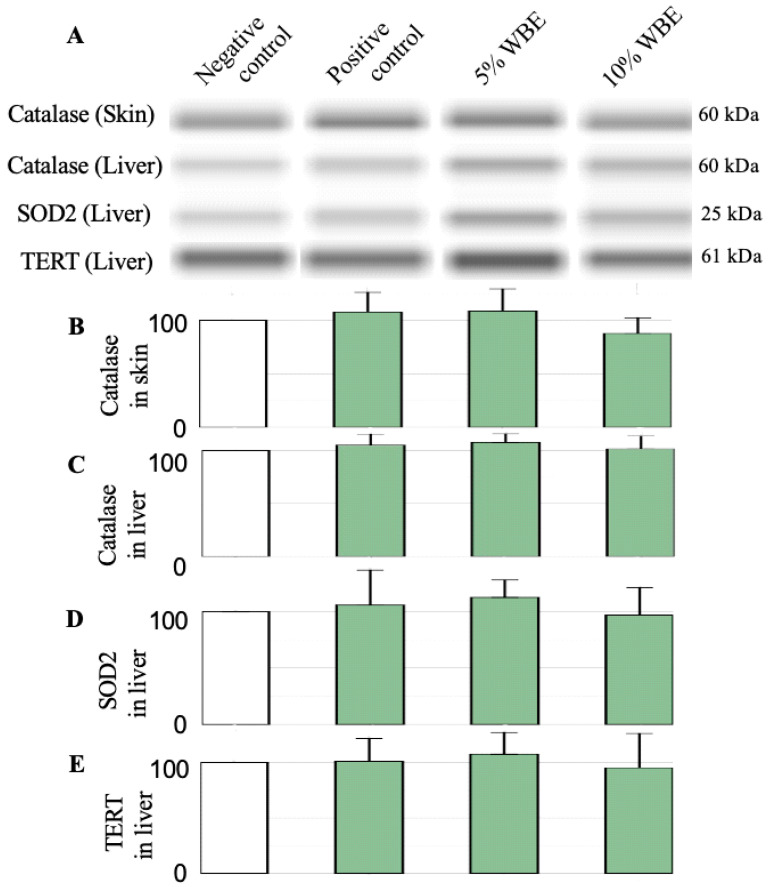
Effect of WBE treatment on protein expressions of catalase, SOD2, and TERT in the skin and livers of galactose-induced accelerated aging rats. (**A**) The protein expression images of catalase, SOD2, and TERT by western blot. (**B**–**E**) The saline-injected negative control group was set as the baseline (100%), and all other values were expressed as percentages relative to this group. Data are presented as mean ± SD (*n* = 10). Negative control, Saline-injected negative control group; Positive control, D-galactose-injected positive control group; 5% WBE, D-galactose-injected with 5% Wheat bran extract diets; 10% WBE, D-galactose-injected with 10% Wheat bran extract diets.

Western blot analysis was performed to assess the protein expression levels of key aging biomarkers (SOD2, catalase, and TERT) in skin and liver tissues (Figure 5). No significant differences were observed between groups, indicating that WBE’s effect may be limited to enzymatic activity rather than transcriptional or translational regulation of these proteins. This suggests that while WBE influences oxidative stress markers in circulation, its impact at the tissue level may be more nuanced and influenced by additional regulatory mechanisms.

## 4. Discussion

This study evaluated the potential of novel antioxidant-enriched wheat bran extracts to counteract aging-related effects, using a D-galactose-induced aging model. The primary outcome of this study demonstrated that oral administration of WBE attenuates D-galactose-induced oxidative stress and cellular senescence in a rat model of accelerated aging, potentially through modulation of endogenous antioxidant defense mechanisms, as hypothesized. The focus was placed on recently identified bioactive compounds in wheat bran, including xylo-oligosaccharides and protein hydrolysates, and their influence on oxidative stress and cellular aging markers. The data demonstrate that dietary WBE supplementation significantly enhanced erythrocyte SOD activity and reduced baseline levels of SA-β-gal, indicating a systemic antioxidative and anti-senescence effect. However, no significant changes were observed in hepatic expression of SOD2, catalase, or telomerase reverse transcriptase (TERT), suggesting that the antioxidative impact of WBE is more pronounced in the circulatory system than in liver and skin tissues.

D-galactose, a naturally occurring monosaccharide, is widely used in gerontological models because it can generate oxidative stress and cause low-grade chronic inflammation when administered over a long period of time [18]. Metabolism of D-galactose by galactose oxidase yields reactive species, notably hydrogen peroxide (H_2_O_2_), which initiates oxidative damage and activates redox-sensitive signaling cascades that are involved in mitochondrial dysfunction and cellular senescence [19]. SA-β-gal, a canonical histochemical marker for senescence, reflects increased lysosomal β-galactosidase activity in acidic environments, a phenotype strongly associated with oxidative stress-induced aging. The observed reduction in SA-β-gal activity in WBE-treated cohorts mechanistically aligns with the attenuation of ROS burden and supports the hypothesis of WBE-mediated cryoprotection.

At the molecular level, D-galactose-induced oxidative stress is known to activate both the p53–p21 and p16INK4a–Rb tumor suppressor pathways, resulting in cell cycle arrest and the maintenance of a senescent state [23]. Furthermore, it has been demonstrated that mitochondrial dysfunction and NADPH oxidase activation contribute to sustained ROS generation and inflammatory signaling [19]. The attenuation of SA-β-gal activity observed in this study may therefore reflect WBE’s capacity to interfere with these canonical senescence-inducing pathways, either by direct antioxidant action or by secondary modulation of inflammatory and mitochondrial responses.

The dose-dependent restoration of erythrocyte SOD activity, particularly notable at 5% and 10% WBE, represents a significant finding. Erythrocytes are terminally differentiated cells that lack nuclei and mitochondria, and thus cannot synthesize new proteins. [24]. Therefore, antioxidant enzymes, such as SOD, cannot increase the amount. Thus, the observed enhancement in SOD activity highlights the systemic bio-efficacy of WBE in strengthening antioxidant defenses. This suggests that WBE supplementation, especially at higher concentrations, may bolster the body’s resilience against oxidative stress. These results align with previous studies attributing potent radical-scavenging properties to phenolic compounds and flavonoids present in wheat bran [25]. Additionally, the reduction in SA-β-gal activity in WBE-treated groups, even under D-galactose-induced stress, indicates a protective effect against cellular senescence [26]. This effect may be mediated through improved mitochondrial homeostasis and a lowered oxidative burden [17,26].

Despite these systemic effects, tissue-level analysis revealed no significant modulation of SOD2, catalase, or TERT protein expression in the liver or skin. This discrepancy may be attributed to several factors: (i) erythrocytes, being terminally differentiated and directly exposed to plasma ROS, may respond more rapidly to exogenous antioxidants, while tissue adaptation may necessitate prolonged exposure [27,28]; (ii) endogenous antioxidant defense systems in the liver may obscure subtle exogenous effects [29,30]; and (iii) the six-week intervention period may have been insufficient to elicit transcriptional or translational changes in solid tissues [31,32,33,34,35] (Table 2). Future studies should consider longer intervention durations and multi-organ profiling to determine the kinetic and spatial characteristics of WBE’s bioactivity.

In terms of metabolic impact, WBE supplementation yielded intriguing effects on body composition. Notably, the D-galactose-treated positive control group displayed a significant reduction in body fat percentage relative to other groups (Figure 2B), consistent with established metabolic perturbations induced by galactose. D-galactose is metabolized via the Leloir pathway, involving enzymes such as galactokinase (GALK), galactose-1-phosphate uridylyl transferase (GALT), and UDP-galactose 4-epimerase (GALE), which convert galactose into glucose for glycolysis or glycogenesis [36]. When D-galactose exceeds metabolic capacity, it disrupts glucose metabolism through competitive inhibition of glucose transporters such as GLUT1 and GLUT4, reducing glucose availability, altering fat storage, and impairing insulin signaling [37,38]. These disruptions contribute to insulin resistance, metabolic dysregulation, and may predispose to age-related pathologies, including neurodegenerative diseases [39,40].

Importantly, WBE supplementation did not induce any adverse effects. Body weight and liver enzyme levels remained unchanged, supporting the safety and tolerability of WBE at the administered dosages. These results suggest that WBE could be considered a safe dietary intervention for mitigating oxidative stress and promoting healthy aging.

Although extrapolation from animal models to humans requires caution, oxidative stress pathways are highly conserved across species. Several components of WBE have been linked to gut health, immune modulation, and antioxidant activity in humans, supporting translational plausibility. Moreover, WBE is a nutritionally valuable but underutilized by-product of cereal processing. Repurposing it as a functional dietary supplement aligns with sustainable agricultural practices while addressing age-related health challenges.

In this study, WBE supplementation resulted in significant improvements in erythrocyte SOD activity and SA-β-gal levels, suggesting potential anti-aging effects in a D-galactose-induced oxidative stress model. However, several other variables-including hepatic SOD2, catalase, and TERT expression-did not show statistically significant differences among groups.

One possible explanation for these non-significant outcomes is the relatively short intervention duration [41]. The six-week treatment period may have been insufficient to elicit measurable changes in tissue-level gene expression or enzymatic activity [41]. Previous studies using the D-galactose-induced aging model have shown that oxidative stress-related gene expression changes often require for 28 weeks of continuous exposure to become detectable [42]. Therefore, future studies should consider extending the intervention period to more fully capture the physiological effects of WBE.

It is also important to note that the absence of statistical significance does not necessarily imply a lack of biological relevance. Subtle shifts in molecular markers may contribute to cumulative benefits over time, even if they fall below the threshold of significance in short-term studies. These findings highlight the need for longitudinal designs and multi-organ profiling to comprehensively elucidate the anti-aging potential of WBE. A limitation of the present study is that blinding was not implemented at any stage.

**Table 2:** Galactose loading vs. SOD activity: erythrocytes vs. liver.

Characteristics	Erythrocyte	Liver
**ROS generation source**	Systemic oxidative stress due to galactose loading [43].	ROS (predominantly of mitochondrial origin) resulting from galactose metabolism [32].
**Type of SOD**	Mainly Cu/Zn-SOD (cytosolic type) [44].	Mn-SOD (mitochondrial type) and Cu/Zn-SOD [45].
**Antioxidant effect**	The direct action of antioxidants is easily observed [27].	Antioxidant effects may be gradual or sub-marked [46].
**The time scale of impact**	Short-term changes in oxidative stress are likely to be reflected [47].	Long-term metabolic compensatory effects may weaken the impact [48].
**Changes in SOD activity**	Often significantly increased or stabilized by antioxidants [49].	Baseline SOD activity is high, and changes may be small [30].
**Effect of damage**	Oxidative stress-induced membrane damage and shortened erythrocyte lifespan are reduced [47].	Limited effect of antioxidants if oxidative stress is too high [50].

## 5. Conclusions

In summary, this study demonstrates that wheat bran extract exhibits promising anti-aging potential by alleviating D-galactose-induced oxidative stress in a rat model. WBE supplementation significantly restored erythrocyte SOD activity and reduced circulating markers of cellular senescence, suggesting enhanced systemic antioxidant defense. Although the impact of WBE on tissue-level aging markers, such as hepatic antioxidant enzymes and telomerase expression, was limited, the observed systemic effects underscore its potential as a functional dietary intervention. These findings support the further development of wheat bran-derived nutraceuticals aimed at promoting healthy aging. Future studies should explore longer intervention durations, higher doses, and underlying molecular pathways to fully elucidate WBE’s role in age-related physiological regulation.

## Figures and Tables

**Table 1:** Composition of ANG 93G and WBE diet.

	AIN-93G	5% WBE	10% WBE
**Composition (%)**			
Carbohydrate	60.1	59.2	58.8
Protein	17.7	17.5	17.4
Fat	7.2	7.1	7.0
**Ingredients (g/kg)**			
Casein	200	187	176
L-Cystine	3	3	3
Corn Starch	397.5	397.5	361.0
Maltodextrin	132	132	132
Sucrose	100	100	100
Soybean Oil	70	69	68.5
Cellulose	50	35	12
Mineral Mix S10022G	35	35	35
Vitamin Mix V10037	10	10	10
Choline Bitartrate	2.5	2.5	2.5
TBHQ, an antioxidant	0.014	0.014	0.014
Wheat Bran Extracts	0	50	100

## Data Availability

The original contributions presented in the study are included in the article, further inquiries can be directed to the corresponding author/s.

## Abbreviations

The following abbreviations are used in this manuscript:

WBE	Wheat Bran Extract
SOD	Superoxide Dismutase
TERT	Telomerase Reverse Transcriptase
SA-β-gal	Senescence-Associated β-Galactosidase
ROS	Reactive Oxygen Species

## References

[1] Guo J., Huang X., Dou L., Yan M., Shen T., Tang W., Li J. (2022). Aging and Aging-Related Diseases: From Molecular Mechanisms to Interventions and Treatments. Signal Transduct. Target. Ther..

[2] Tenchov R., Sasso J.M., Wang X., Zhou Q.A. (2023). Aging Hallmarks and Progression and Age-Related Diseases: A Landscape View of Research Advancement. ACS Chem. Neurosci..

[3] Yang J., Luo J., Tian X., Zhao Y., Li Y., Wu X. (2024). Progress in Understanding Oxidative Stress, Aging, and Aging-Related Diseases. Antioxidants.

[4] Shi S., Wang L., van der Laan L.J.W., Pan Q., Verstegen M.M.A. (2021). Mitochondrial Dysfunction and Oxidative Stress in Liver Transplantation and Underlying Diseases: New Insights and Therapeutics. Transplantation.

[5] Rossiello F., Jurk D., Passos J.F., d’Adda di Fagagna F. (2022). Telomere Dysfunction in Ageing and Age-Related Diseases. Nat. Cell Biol..

[6] Grodstein F., Yu L., de Jager P.L., Levey A., Seyfried N.T., Bennett D.A. (2022). Exploring Cortical Proteins Underlying the Relation of Neuroticism to Cognitive Resilience. Aging Brain.

[7] Dutta C. (2024). BIOMARKERS OF AGING. Innov. Aging.

[8] Tao W., Yu Z., Han J.-D.J. (2024). Single-Cell Senescence Identification Reveals Senescence Heterogeneity, Trajectory, and Modulators. Cell Metab..

[9] Arbatskiy M.S., Balandin D.E. (2024). Universal Markers of Cellular and Replicative Senescence. Adv. Gerontol..

[10] Palma F.R., He C., Danes J.M., Paviani V., Coelho D.R., Gantner B.N., Bonini M.G. (2020). Mitochondrial Superoxide Dismutase: What the Established, the Intriguing, and the Novel Reveal About a Key Cellular Redox Switch. Antioxid. Redox Signal.

[11] Weyemi U., Parekh P.R., Redon C.E., Bonner W.M. (2012). SOD2 Deficiency Promotes Aging Phenotypes in Mouse Skin. Aging.

[12] Rasheed Z. (2024). Therapeutic Potentials of Catalase: Mechanisms, Applications, and Future Perspectives. Int. J. Health Sci..

[13] Anwar S., Alrumaihi F., Sarwar T., Babiker A.Y., Khan A.A., Prabhu S.V., Rahmani A.H. (2024). Exploring Therapeutic Potential of Catalase: Strategies in Disease Prevention and Management. Biomolecules.

[14] Qu H., Madl R.L., Takemoto D.J., Baybutt R., Wang W. (2005). Phytochemical Lignans Associated with the Cancer Prevention by Wheat Bran. J. Nutr..

[15] Rahman M.S., Qi G., Li C., Li Y., Wang W., Atala A., Sun X.S. (2025). Differential Effects of Wheat Bran Antioxidants on the Growth Dynamics of Human Cancer Cells. Foods.

[16] Li C., Wu W., Tilley M., Chen R., Sun X.S., Wang W., Li Y. (2024). In Vitro Antioxidant Properties of Wheat Bran Extracts and Their Inhibitory Effects on Collagenase, Elastase, and Hyaluronidase. ACS Food Sci. Technol..

[17] Kobayashi K., Suzauddula M., Bender R., Li C., Li Y., Sun X.S., Wang W. (2025). Functional Properties and Potential Applications of Wheat Bran Extracts in Food and Cosmetics: A Review of Antioxidant, Enzyme-Inhibitory, and Anti-Aging Benefits. Foods.

[18] Pantiya P., Thonusin C., Ongnok B., Chunchai T., Kongkaew A., Nawara W., Arunsak B., Chattipakorn N., Chattipakorn S.C. (2023). Chronic D-Galactose Administration Induces Natural Aging Characteristics, in Rat’s Brain and Heart. Toxicology.

[19] Guo B., Guo Q., Wang Z., Shao J.-B., Liu K., Du Z.-D., Gong S.-S. (2020). D-Galactose-Induced Oxidative Stress and Mitochondrial Dysfunction in the Cochlear Basilar Membrane: An in Vitro Aging Model. Biogerontology.

[20] Bruce C.L., Juszczak E., Ogollah R., Partlett C., Montgomery A. (2022). A Systematic Review of Randomisation Method Use in RCTs and Association of Trial Design Characteristics with Method Selection. BMC Med. Res. Methodol..

[21] Zhang H., Chen C., Liu Y., Chen W., Qi J., Xu Y., Ren L., Yang G., Min D., Liu Z. (2023). D-Galactose Causes Sinoatrial Node Dysfunction: From Phenotype to Mechanism. Aging.

[22] Zheng M., Liu Y., Zhang G., Yang Z., Xu W., Chen Q. (2023). The Applications and Mechanisms of Superoxide Dismutase in Medicine, Food, and Cosmetics. Antioxidants.

[23] Huang W., Hickson L.J., Eirin A., Kirkland J.L., Lerman L.O. (2022). Cellular Senescence: The Good, the Bad and the Unknown. Nat. Reviews. Nephrol..

[24] Ghaffari S. (2008). Oxidative Stress in the Regulation of Normal and Neoplastic Hematopoiesis. Antioxid. Redox Signal.

[25] Brewer L.R., Kubola J., Siriamornpun S., Shi Y.-C. (2024). Distribution of Antioxidants and Phenolic Compounds in Flour Milling Fractions from Hard Red Winter Wheat. Grain Oil Sci. Technol..

[26] Homolak J., Varvaras K., Sciacca V., Babic Perhoc A., Virag D., Knezovic A., Osmanovic Barilar J., Salkovic-Petrisic M. (2024). Insights into Gastrointestinal Redox Dysregulation in a Rat Model of Alzheimer’s Disease and the Assessment of the Protective Potential of D-Galactose. ACS Omega.

[27] Möller M.N., Orrico F., Villar S.F., López A.C., Silva N., Donzé M., Thomson L., Denicola A. (2023). Oxidants and Antioxidants in the Redox Biochemistry of Human Red Blood Cells. ACS Omega.

[28] Eigenschink M., Savran D., Zitterer C.P., Granitzer S., Fritz M., Baron D.M., Müllner E.W., Salzer U. (2021). Redox Properties of Human Erythrocytes Are Adapted for Vitamin C Recycling. Front. Physiol..

[29] Zhu J., Lian J., Wang X., Wang R., Pang X., Xu B., Wang X., Li C., Ji S., Lu H. (2023). Role of Endogenous and Exogenous Antioxidants in Risk of Six Cancers: Evidence from the Mendelian Randomization Study. Front. Pharmacol..

[30] Trist D.B.G., Hilton D.J.B., Hare P.D.J., Crouch P.P.J., Double P.K.L. (2020). Superoxide Dismutase 1 in Health and Disease: How a Frontline Antioxidant Becomes Neurotoxic. Angew. Chem. (Int. Ed. Engl.).

[31] Remigante A., Morabito R., Spinelli S., Trichilo V., Loddo S., Sarikas A., Dossena S., Marino A. (2020). D-Galactose Decreases Anion Exchange Capability through Band 3 Protein in Human Erythrocytes. Antioxidants.

[32] Allameh A., Niayesh-Mehr R., Aliarab A., Sebastiani G., Pantopoulos K. (2023). Oxidative Stress in Liver Pathophysiology and Disease. Antioxidants.

[33] Ross K.S., Smith C. (2020). D-Galactose: A Model of Accelerated Ageing Sufficiently Sensitive to Reflect Preventative Efficacy of an Antioxidant Treatment. Biogerontology.

[34] Homolak J., Babic Perhoc A., Knezovic A., Osmanovic Barilar J., Virag D., Joja M., Salkovic-Petrisic M. (2021). The Effect of Acute Oral Galactose Administration on the Redox System of the Rat Small Intestine. Antioxidants.

[35] Zhao D., Liu X., Zhao S., Li Z., Qin X. (2021). 1H NMR-Based Fecal Metabolomics Reveals Changes in Gastrointestinal Function of Aging Rats Induced by d-Galactose. Rejuvenation Res..

[36] Nagode A., Vanbeselaere J., Dutkiewicz Z., Kaltenbrunner S., Wilson I.B.H., Duchêne M. (2023). Molecular Characterisation of Entamoeba Histolytica UDP-Glucose 4-Epimerase, an Enzyme Able to Provide Building Blocks for Cyst Wall Formation. PLoS Neglected Trop. Dis..

[37] Vargas E., Podder V., Carrillo Sepulveda M.A. (2025). Physiology, Glucose Transporter Type 4. StatPearls.

[38] Chadt A., Al-Hasani H. (2020). Glucose Transporters in Adipose Tissue, Liver, and Skeletal Muscle in Metabolic Health and Disease. Pflug. Arch..

[39] Shwe T., Pratchayasakul W., Chattipakorn N., Chattipakorn S.C. (2018). Role of D-Galactose-Induced Brain Aging and Its Potential Used for Therapeutic Interventions. Exp. Gerontol..

[40] Lin Y., Wang Y., Yang X., Ding Z., Hu M., Huang X., Zhang Q., Yu Y. (2025). NMN Reverses D-Galactose-Induced Neurodegeneration and Enhances the Intestinal Barrier of Mice by Activating the Sirt1 Pathway. Front. Pharmacol..

[41] Cardoso A., Magano S., Marrana F., Andrade J.P. (2015). D-Galactose High-Dose Administration Failed to Induce Accelerated Aging Changes in Neurogenesis, Anxiety, and Spatial Memory on Young Male Wistar Rats. Rejuvenation Res..

[42] Pantiya P., Thonusin C., Ongnok B., Chunchai T., Kongkaew A., Nawara W., Arunsak B., Chattipakorn N., Chattipakorn S.C. (2023). Long-Term D-Galactose Administration Mimics Natural Aging in Rat’s Hippocampus. Alzheimer’s Dement..

[43] Bettiol A., Galora S., Argento F.R., Fini E., Emmi G., Mattioli I., Bagni G., Fiorillo C., Becatti M. (2022). Erythrocyte Oxidative Stress and Thrombosis. Expert. Rev. Mol. Med..

[44] Toro-Román V., Siquier-Coll J., Bartolomé I., Grijota F.J., Muñoz D., Maynar-Mariño M. (2021). Copper Concentration in Erythrocytes, Platelets, Plasma, Serum and Urine: Influence of Physical Training. J. Int. Soc. Sports Nutr..

[45] Kitada M., Xu J., Ogura Y., Monno I., Koya D. (2020). Manganese Superoxide Dismutase Dysfunction and the Pathogenesis of Kidney Disease. Front. Physiol..

[46] Radosavljevic T., Brankovic M., Djuretić J., Grujic-Milanovic J., Kovacic M., Jevtic J., Stankovic S., Samardzic J., Vucevic D., Jakovljevic V. (2025). Alpinetin Exhibits Antioxidant and Anti-Inflammatory Effects in C57BL/6 Mice with Alcoholic Liver Disease Induced by the Lieber–DeCarli Ethanol Liquid Diet. Int. J. Mol. Sci..

[47] Obeagu E.I., Igwe M.C., Obeagu G.U. (2024). Oxidative Stress’s Impact on Red Blood Cells: Unveiling Implications for Health and Disease. Medicine.

[48] Li S., Tan H.-Y., Wang N., Zhang Z.-J., Lao L., Wong C.-W., Feng Y. (2015). The Role of Oxidative Stress and Antioxidants in Liver Diseases. Int. J. Mol. Sci..

[49] Ściskalska M., Ołdakowska M., Marek G., Milnerowicz H. (2020). Changes in the Activity and Concentration of Superoxide Dismutase Isoenzymes (Cu/Zn SOD, MnSOD) in the Blood of Healthy Subjects and Patients with Acute Pancreatitis. Antioxidants.

[50] Vascotto C., Tiribelli C., Albano E., Parola M. (2015). Oxidative Stress, Antioxidant Defenses, and the Liver. Studies on Hepatic Disorders.

